# Prognostic significance of preoperative systemic inflammatory biomarkers in patients with hepatocellular carcinoma after microwave ablation and establishment of a nomogram

**DOI:** 10.1038/s41598-021-93289-3

**Published:** 2021-07-05

**Authors:** Shuai Wang, Yan Deng, Xiao Yu, Xue-Wen Zhang, Cheng-Long Huo, Zhen-Gang Sun, Hong Chang

**Affiliations:** 1 Department of Hepatobiliary Surgery, Shandong Provincial Hospital, Cheeloo College of Medicine, Shandong University, No. 9677, Jingshi Road, Lixia District, Jinan, 500212 Shandong China; 2 Department of Hepatobiliary Surgery, Jing Zhou Central Hospital, The Second Clinical Medical College, Yangtze University, Jing Zhou, 434020 Hubei China

**Keywords:** Cancer, Biomarkers, Diseases, Oncology, Risk factors

## Abstract

The study aimed to evaluate the prognostic significance of preoperative systemic inflammatory biomarkers including albumin to globulin ratio (AGR), neutrophil to lymphocyte ratio (NLR), lymphocyte to monocyte ratio (LMR), and platelet to lymphocyte ratio (PLR) and establish a nomogram in hepatocellular carcinoma (HCC) patients after microwave ablation (MWA). 192 HCC patients receiving MWA as initial therapy from the first ward of hepatobiliary surgery were classified as training cohort. Whereas, 84 patients from the second of hepatobiliary surgery were classified as validation cohort. Kaplan–Meier (KM) method and univariate analyses showed that AGR, NLR, LMR, and PLR were significantly associated with OS in the training cohort. Multivariate analysis including clinicopathologic features screened out independent predictors including ascites, tumor size, cancer embolus, AGR, and PLR. Based on those variables, a nomogram for predicting OS was established. The C-index was 0.794 in the training cohort and 0.772 in the validation cohort. Calibration plots identified the nomogram performed well with an ideal model. Compared with Barcelona Clinic Liver Cancer (BCLC) staging system and simple tumor size, the nomogram showed better predictive ability. Besides, the nomogram discovered the highest diagnostic accuracy in predicting postoperative clinical outcome than the combination of the present models with tumor size. In conclusion, the constructed nomogram could accurately predict individualized survival probability and might support clinician in individual treatment optimization and clinical decision-making.

## Introduction

Hepatocellular carcinoma (HCC) ranks sixth most lethal malignancies globally, which attracts significant attention due to its aggressive biologic behavior and poor clinical prognosis ^1,2^. Now radical resection, liver transplantation and microwave ablation (MWA) are the main curative treatments^3,4^. Due to multifocal node or poor liver function resulting from severe cirrhosis, many patients have missed the chance of complete resection^5^. Liver transplantation may be another option for those patients, but the surgery is often unfeasible because of the shortage of donors^6^. Therefore, ablation may be a unique radical treatment for those patients, though HCC patients after ablation has been reported at higher 5-year recurrence rate and equal 5-year overall survival (OS) time compared to those after radical resection or liver transplantation^7,8^.

Despite an improving progress in the multimodal therapy, the survival benefit remain to be limited^9^. Hence, there is a need to discover appropriate markers for evaluating prognosis to achieve precise individualized therapy in HCC patients. Nomogram established based on multiple independent risk factors is a statistical model with a high reliability.

Recently, it’s suggested that systemic inflammatory response functions as a vital role in initiation, progression, metastasis, and treatment resistance of malignancy tumor and then it might correlate with worse prognosis^10,11^. What is more, systemic inflammatory response of malignancy patients could be presented by the changing of peripheral blood cell counts, including neutrophil, lymphocyte, monocyte, platelet, globulin and so on^12^. Based on those cell counts, inflammatory response markers including albumin to globulin ratio (AGR), neutrophil to lymphocyte ratio (NLR), lymphocyte to monocyte ratio (LMR), and platelet to lymphocyte ratio (PLR) have been proven to be independently prognostic factors in various malignancy^13–15^. Published studies have revealed that NLR, LMR, and PLR might be correlated with outcome in HCC patients after microwave ablation^16–18^. To the best of our knowledge, few studies evaluated the associations of all the inflammatory markers including AGR with prognosis and no more relevant nomogram was established among HCC patients after MWA.

Hence, the present aimed to evaluate the prognostic significance of preoperative AGR, NLR, LMR and PLR and establish a prognostic nomogram to predict OS in HCC patients after MWA.

## Results

### Clinicopathological characteristics

**Figure 1:**
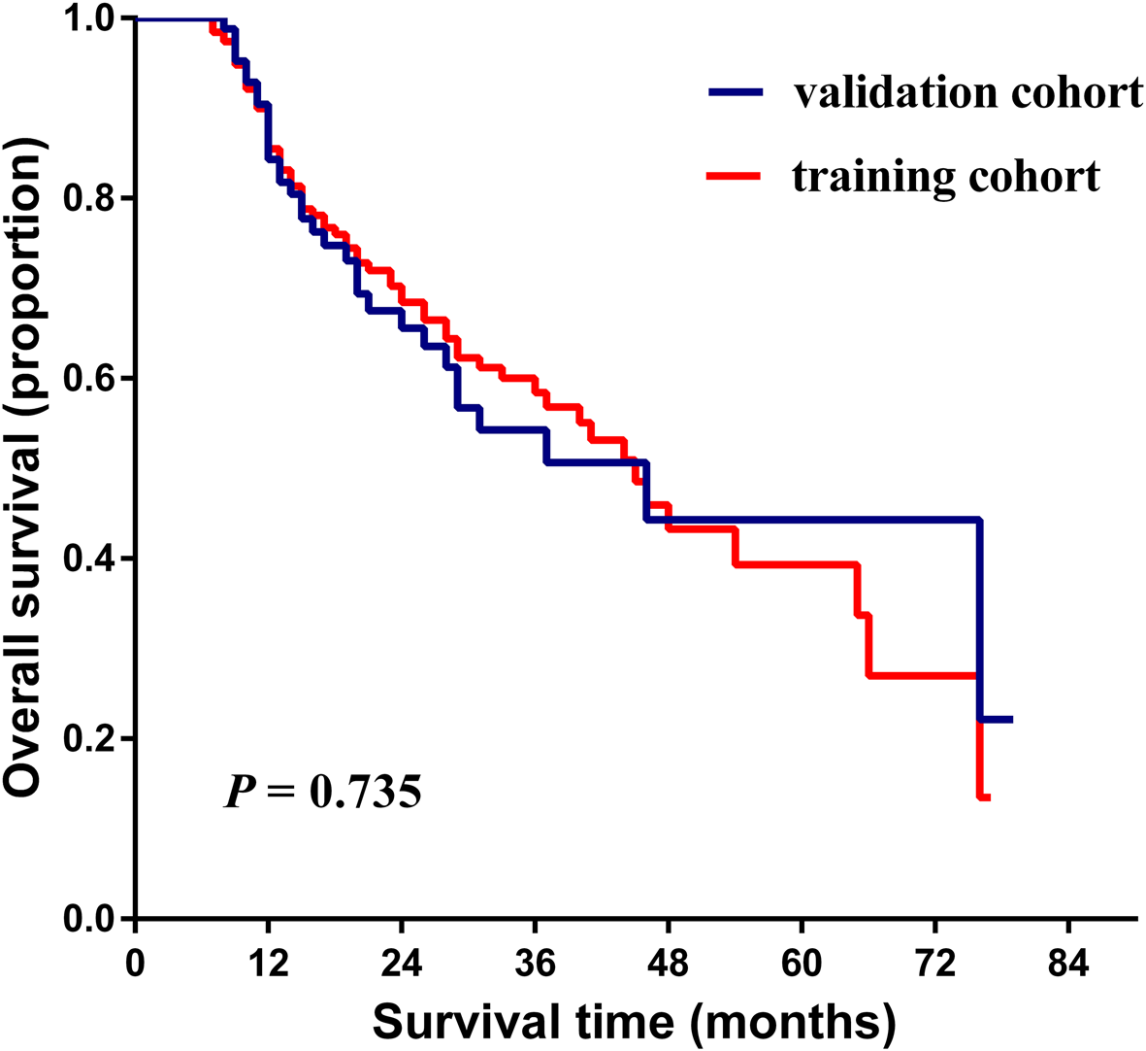
Kaplan–Meier estimate of the postoperative overall survival (OS) of patients with HCC stratified by different cohorts. The figure was performed using R software (version 3.3.1, https://www.r-project.org/).

Baseline clinicopathological characteristics of 276 patients (194 in the training group and 84 in the validation group) were summarized in Table 1. All the clinicopathological variables excepting cancer embolus were matched between two cohorts (*P* > 0.050). Compared with the validation group, the presence of cancer embolus in the training cohort was significantly less. The median follow-up time was 37.5 months (range: 6.0–77.0 months) for the training group and 38.5 (range: 6.0–79.0 months) for the validation group. Median time of OS was 18 months (range 6–79 months). As showed in Fig. 1, the 1-, 3-, and 5-year OS rates were 85.50%, 58.40%, and 39.30% for the training cohort, 84.30%, 54.30% and 44.30% for the validation cohort, respectively. KM curves showed there was no significant difference in overall survival time between two cohorts (*P* > 0.050).

**Table 1:** Baseline demographics and clinical characteristics of patients in training cohort and validation cohort.

Variable	Training cohort (n = 192) n/median (range)	Validation cohort (n = 84) n/median (range)	*P* value
Age (years)	55 (29–83)	53.5 (27–81)	0.209
Gender (male/female)	145/49	64/20	0.797
Hypertension (present/absent)	33/159	14/70	0.916
Diabetes (present/absent)	21/171	10/74	0.815
Smoking (present/absent)	53/139	24/60	0.869
Drinking (present/absent)	40/152	23/61	0.233
Liver cirrhosis (present/absent)	123/69	53/31	0.878
Ascites (present/absent)	19/173	9/75	0.836
HBV infection (present/absent)	143/49	61/23	0.746
HCV infection (present/absent)	23/169	12/72	0.596
AFP (ng/ml)	32.30 (1.15–105,184.00)	26.83 (1.25–20,320.00)	0.359
Child–Pugh (A/B)	166/26	68/16	0.241
Tumor size (< 3/ ≥ 3) (cm)	158/34	70/24	0.122
Tumor number (1/ > 1)	146/46	56/28	0.106
BCLC stage (A/B/C/D)	24/120/23/25	6/45/17/16	0.09
Cancer embolus (present/absent)	10/182	16/68	** < 0.001**
Tumor capsule (intact/incomplete)	136/56	56/28	0.234
Serum albumin (g/L)	36.95 (21.79–49.30)	36.525 (25.40–49.54)	0.685
Globulin (g/L)	29.23 (8.80–52.02)	28.90 (16.91–41.20)	0.144
Lymphocyte (k/mm^2^)	1.08 (0.08–3.48)	1.22 (0.34–3.27)	0.184
Monocyte (k/mm^2^)	0.32 (0.01–3.13)	0.36 (0.02–1.39)	0.915
Platelet (k/mm^2^)	100.00 (34.00–408.00)	113.00 (49.00–207.00)	0.128
Neutrophil (k/mm^2^)	2.32 (0.52–17.70)	2.44 (0.80–16.30)	0.446

*P* value of less than 0.050 was considered to indicate significant difference.

*AFP* alpha-fetoprotein, *HBV* hepatitis B virus, *HCV* hepatitis C virus, *BCLC* Barcelona clinic liver cancer.

### Time ROC curve analysis in training cohort

**Figure 2:**
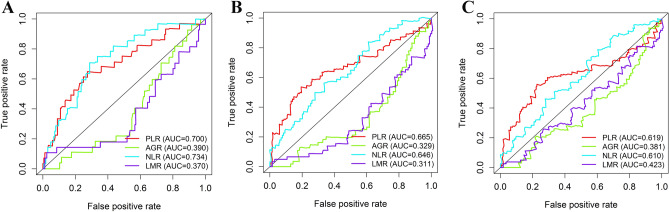
Comparison of the predictive ability of systemic inflammatory biomarkers in predicting 1- **(A)**, 3- **(B)**, and 5-year **(C) **survival. The figure was performed using R software (version 3.3.1, Vienna, Austria, https://www.r-project.org/).

Time ROC curve analysis to explore the predictive value of systemic inflammatory biomarkers. As shown in Fig. 2, PLR, AGR, NLR, and LMR showed the well predictive ability in predicting 1- (AUC: 0.610 for AGR, 0.734 for NLR, and 0.630 for LMR, 0.700 for PLR), 3- (AUC: 0.671 for AGR, 0.646 for NLR, and 0.689 for LMR, 0.665 for PLR), and 5-year (AUC: 0.619 for AGR, 0.610 for NLR, and 0.577 for LMR, 0.619 for PLR) survival, respectively.

### Associations of biomarkers with clinicopathological features in training cohort

Detail associations of AGR, NLR, LMR, and PLR with baseline clinicopathological factors were assessed in Table 2. Obviously, AGR was correlated to liver cirrhosis and ascites. Tumor size was significantly associated with NLR, PLR, and LMR, whereas high PLR and low LMR were prone to appear in patients with cancer embolus.

**Table 2:** Associations of inflammation-based markers with clinicopathologic characteristics in training cohort.

Variables	AGR		NLR		LMR		PLR	
	Median(range)	*P* value	Median(range)	*P* value	Median(range)	*P* value	Median(range)	*P* value
Total	1.252 (0.517–4.943)		1.907 (0.321–19.833)		3.484 (0.026–168.000)		86.307 (16.667–962.500)	
**Age(years)**		0.335		0.945		0.867		0.997
< 60	1.324 (0.619–4.943)		1.907 (0.321–19.833)		3.455 (0.620–168.000)		85.965 (16.667–413.115)	
≥ 60	1.188 (0.517–2.086)		1.907 (0.615–16.729)		3.566 (0.256–27.500)		88.763 (27.211–962.500)	
**Gender**		0.766		0.317		0.361		0.886
Male	1.257 (0.517–4.943)		1.952 (0.321–16.729)		3.451 (0.620–168.000)		86.632 (16.667–413.115)	
Female	1.244 (0.668–2.114)		1.874 (0.500–19.833)		3.682 (0.026–66.5000)		85.965 (32.727–962.500)	
**Hypertension**		0.808		0.557		0.679		0.169
Present	1.324 (0.517–4.943)		1.935 (0.616–10.599)		3.720 (0.026–27.500)		80.769 (33.921–962.500)	
Absent	1.247 (0.619–3.732)		1.890 (0.321–19.833)		3.375 (0.620–168.000)		87.347 (16.667–413.115)	
**Diabetes mellitus**		0.109		0.529		0.469		**0.035**
Present	1.156 (0.648–4.943)		1.812 (0.616–6.758)		3.620 (1.719–7.111)		72.137 (27.211–370.968)	
Absent	1.252 (0.517–3.732)		1.933 (0.321–19.833)		3.374 (0.026–168.000)		89.561 (16.667–962.500)	
**Smoking**		0.217		0.175		0.107		0.575
Present	1.324 (0.745–4.943)		1.925 (0.853–10.599)		3.556 (0.026–168.000)		87.194 (16.667–413.115)	
Absent	1.247 (0.517–3.532)		1.895 (0.321–19.833)		3.447 (1.008–12.429)		85.531 (27.211–962.500)	
**Drinking**		0.631		0.121		0.634		0.668
Present	1.287 (0.754–3.732)		1.931 (0.321–19.833)		3.484 (0.026–168.000)		81.051 (16.667–413.115)	
Absent	1.252 (0.517–4.943)		1.860 (0.690–3.923)		3.382 (1.008–12.429)		88.735 (27.211–962.500)	
**Liver cirrhosis **		**0.010**		0.172		0.545		0.101
Present	1.193 (0.517–3.732)		1.836 (0.321–9.985)		3.616 (0.620–66.500)		84.132 (16.667–392.157)	
Absent	1.384 (0.687–4.943)		2.132 (0.573–19.833)		3.214 (0.026–168.000)		94.286 (32.727–962.500)	
**Ascites**		**0.004**		0.224		0.072		0.954
Present	1.010 (0.687–1.734)		2.000 (0.500–16.729)		2.357 (0.620–27.500)		89.091 (33.508–331.250)	
Absent	1.324 (0.517–4.973)		1.903 (0.321–19.833)		3.566 (0.026–168.000)		86.136 (16.667–962.500)	
**HBV infection**		0.637		**0.040**		0.227		0.263
Present	1.250 (0.517–3.532)		1.849 (0.394–19.833)		3.616 (0.620–168.000)		88.295 (16.667–413.115)	
Absent	1.252 (0.626–4.943)		2.152 (0.321–16.729)		3.159 (0.026–7.111)		81.481 (27.211–962.500)	
**HCV infection**		**0.049**		0.828		0.509		0.701
Present	1.041 (0.626–3.732)		2.152 (0.321–8.750)		3.455 (0.026–6.000)		78.561 (27.211–962.500)	
Absent	1.257 (0.517–4.943)		1.881 (0.394–19.833)		3.492 (0.620–168.000)		87.347 (16.667–413.115)	
**AFP**		0.848		0.243		0.267		0.073
≥ 20 ng/ml	1.314 (0.626–1.966)		1.935 (0.321–10.599)		3.000 (0.620–168.000)		98.765 (16.667–413.115)	
< 20 ng/ml	1.252 (0.517–4.943)		1.882 (0.500–19.833)		3.677 (0.026–66.500)		84.000 (27.211–962.500)	
**Child–Pugh**		0.214		0.098		0.106		0.606
A	1.287 (0.626–4.943)		1.877 (0.321–16.729)		3.571 (0.026–168.000)		85.119 (16.667–962.500)	
B	1.104 (0.517–1.966)		2.704 (0.594–19.833)		2.461 (0.620–27.500)		90.030 (33.508–392.157)	
**Tumor size**		0.783		**0.003**		**0.005**		**0.006**
< 3 cm	1.287 (0.517–4.943)		1.871 (0.321–19.833)		3.712 (0.026–168.000)		83.766 (26.693–962.500)	
≥ 3 cm	1.218 (0.668–2.086)		3.125 (0.853–10.599)		2.408 (0.989–27.500)		118.256 (16.667–413.115)	
**Tumor number**		0.356		0.785		0.271		1.000
1	1.242 (0.517–4.943)		1.920 (0.321–19.833)		3.407 (0.026–66.500)		86.956 (26.693–962.500)	
> 1	1.403 (0.758–3.732)		1.891 (0.853–10.599)		4.121 (0.989–168.000)		83.595 (16.667–253.763)	
**Cancer embolus**		0.224		0.053		**0.030**		**0.004**
Present	1.164 (0.758–1.685)		3.221 (0.954–9.985)		2.185 (0.989–9.429)		118.256 (61.481–413.115)	
Absent	1.287 (0.517–4.943)		1.877 (0.321–19.833)		3.611 (0.026–168.000)		84.000 (16.667–962.500)	
**Tumor capsule**		0.565		0.994		0.608		0.924
Intact	1.277 (0.517–4.943)		1.920 (0.394–19.833)		3.407 (0.620–168.000)		85.098 (16.667–392.157)	
Incomplete	1.235 (0.626–3.732)		1.866 (0.321–16.729)		3.721 (0.026–66.500)		89.545 (26.693–962.500)	

*P* value of less than 0.050 was considered to indicate significant difference. The *P* value was calculated by nonparametric Wilcoxon rank-sum test.

*AFP* alpha-fetoprotein, *HBV* hepatitis B virus, *HCV* hepatitis C virus, *AGR* albumin to globulin ratio, *NLR* neutrophil to lymphocyte ratio, *PLR* platelet to lymphocyte ratio, *LMR* lymphocyte to monocyte ratio.

### Independent risk factors in the training cohort

**Figure 3:**
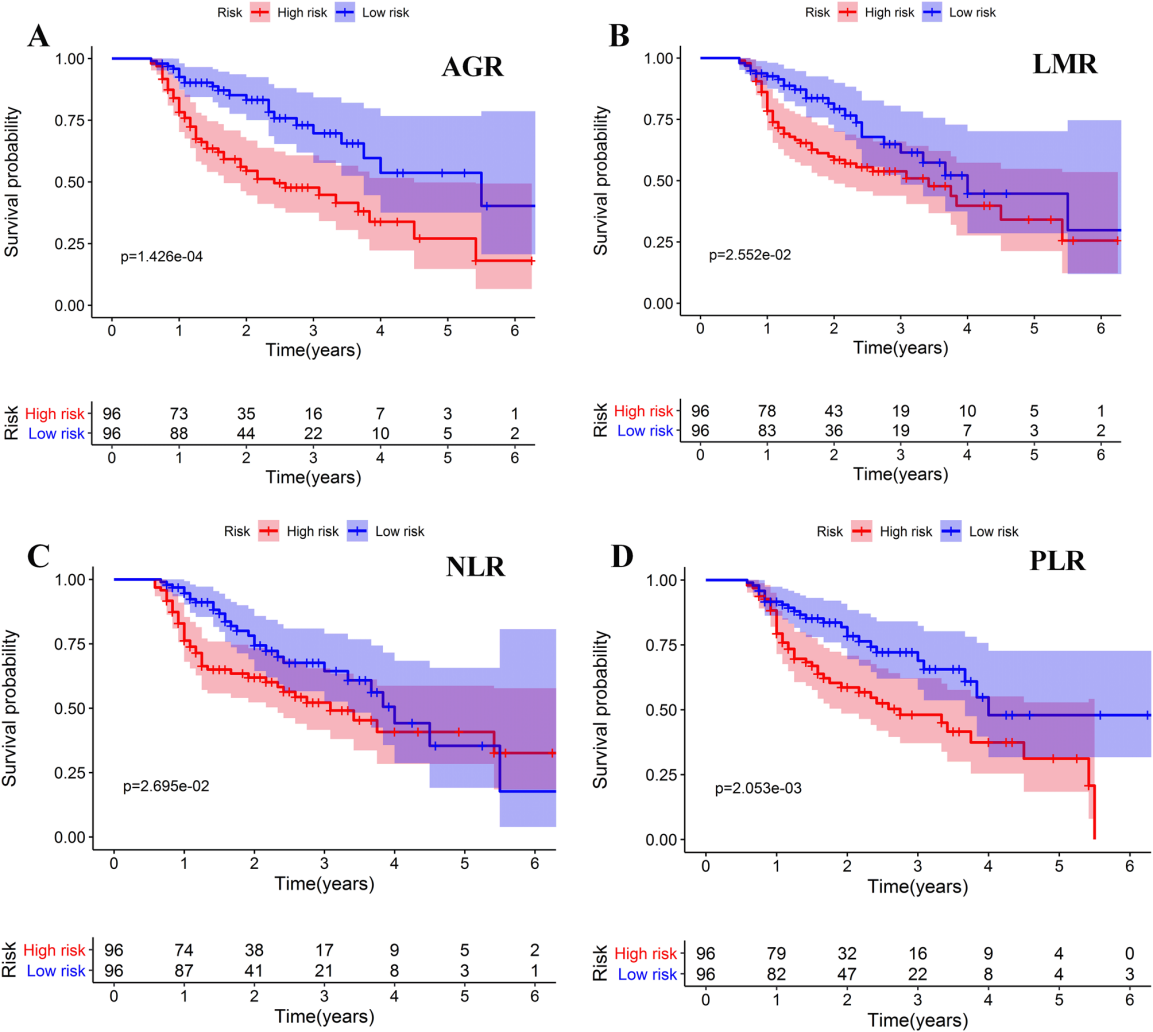
Kaplan–Meier curves for OS stratified by AGR **(A)**, LMR **(B)**, NLR **(C)**, and PLR **(D)**. The figure was performed using R software (version 3.3.1, Vienna, Austria, https://www.r-project.org/).

Based on the median value of systemic inflammatory biomarkers, all the patients were classified into high- and low- risk groups, respectively. As shown in Fig. 3, KM curves confirmed that AGR, NLR, PLR, and LMR were strong predictors of OS. Results from univariate analysis showed that BCLC staging system, ascites, tumor size, cancer embolus. AGR, NLR, LMR, and PLR were significantly indicators. In multivariate analysis, ascites (HR: 2.194; 95% CI: 1.135–4.238; *P* = 0.019), tumor size (HR: 2.201; 95% CI: 1.226–3.950; *P* = 0.008), cancer embolus (HR: 2.372; 95% CI: 1.327–4.238; *P* = 0.004), AGR (HR: 0.465; 95% CI: 0.218–0.991; *P* = 0.047), and PLR (HR: 1.003; 95% CI: 1.001–1.005; *P* = 0.014) were further screened out as independent risk factors for OS (Table 3).

**Table 3:** Univariate and multivariate Cox proportional hazards regression analyses of overall survival in the training cohort.

Variables	Factor	Univariate analysis		Multivariate analysis	
		HR (95% CI)	*P* value	HR (95% CI)	*P* value
Age	≥ 60 vs < 60 (years)	1.408 (0.838–2.366)	0.196		
Gender	Male vs female)	1.155 (0.660–2.023)	0.614		
Hypertension	Present vs absent	0.945 (0.480–1.862)	0.870		
Diabetes	Present vs absent	0.340 (0.106–1.086)	0.069		
Smoking	Present vs absent	0.932 (0.520–1.669)	0.812		
Drinking	Present vs absent	1.035 (0.548–1.955)	0.915		
Liver cirrhosis	Present vs absent	1.188 (0.699–2.020)	0.525		
Ascites	Present vs absent	1.946 (1.036–3.654)	**0.038**	2.194 (1.135–4.238)	**0.019**
HBV infection	Present vs absent	1.247 (0.703–2.211)	0.450		
HCV infection	Present vs absent	0.632 (0.253–1.580)	0.326		
AFP	≥ 20 vs < 20 (ng/ml)	1.441 (0.857–2.424)	0.169		
Child–Pugh	A vs B	1.152 (0.592–2.218)	0.672		
Tumor size	≥ 3 vs < 3(cm)	3.936 (2.380–6.509)	** < 0.001**	2.201 (1.226–3.950)	**0.008**
Tumor number	> 1 vs 1	1.709 (0.835–3.496)	0.142		
Cancer embolus	Present vs absent	3.891 (2.394–6.323)	** < 0.001**	2.372 (1.327–4.238)	**0.004**
Tumor capsule	Intact vs incomplete	1.275 (0.729–2.229)	0.395		
BCLC stage	Per 1 stage increasing	1.785 (1.389–2.295)	** < 0.001**	NA	NA
AGR	Per 1 increasing	0.296 (0.139–0.632)	**0.002**	0.465 (0.218–0.991)	**0.047**
NLR	Per 1 increasing	1.130 (1.061–1.205)	** < 0.001**	1.080 (0.990–1.179)	0.082
LMR	Per 1 increasing	0.949 (0.849–1.061)	0.356		
PLR	Per 1 increasing	1.004 (1.002–1.006)	** < 0.001**	1.003 (1.001–1.005)	**0.014**

*P* value of less than 0.050 was considered to indicate significant difference.

*AFP* alpha-fetoprotein, *HBV* hepatitis B virus, *HCV* hepatitis C virus, *AGR *albumin to globulin ratio, *NLR* neutrophil to lymphocyte ratio, *PLR* platelet to lymphocyte ratio, *LMR* lymphocyte to monocyte ratio, *HR* hazard ratio, *CI* confidence interval.

### Prognostic nomogram for survival

**Figure 4:**
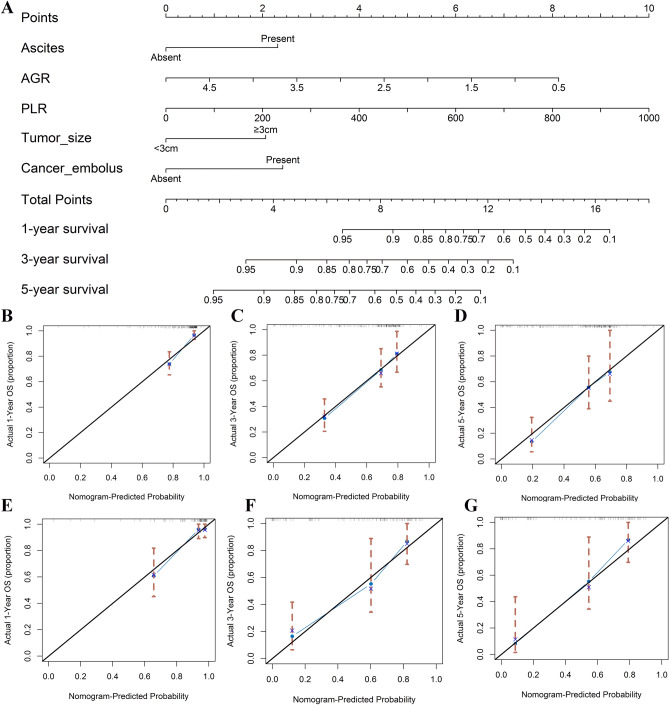
Nomogram for predicting 1-, 3-, and 5-year OS among HCC patients undergoing microwave ablation in the training cohort. The black line represents the “ideal” line of a perfect match between predicted and observed survival. The blue line indicates the performance of the proposed nomogram. The X-axis is nomogram predicted probability of survival and Y-axis is actual survival. Blue dots are sub-cohorts of the data set; red vertical bars represent 95% confidence interval **(A)**. Calibration curves of the nomogram for predicting 1- **(B)**, 3- **(C)**, and 5-year survival **(D)** in the training cohort. Calibration curves of the nomogram for predicting 1-year **(E)**, 3-year **(F)**, and 5-year survival **(G)** in the validation cohort. The figure was performed using R software (version 3.3.1, Vienna, Austria, https://www.r-project.org/).

Based on those independent risk factors, a prognostic nomogram was constructed for OS prediction in the training cohort. Each subgroup variable was assigned a corresponding score for the establishment of the nomogram. The larger points in the nomogram indicated poorer OS. Each variable was assigned a weighted number of points in the nomogram, then the sum of points for each patient was in accordance with a specific 1-, 3-, and 5-year OS rates (Fig. 4A).

### Validation of the constructed nomograms

For internal validation, boot-strapped calibration plots predicting 1-, 3-, and 5-year OS (Fig. 4B–D) performed well with ideal model. Besides, nomograms exhibited good efficacy in estimating postoperative OS with a high C-index of 0.794 in the training cohort.

For external validation in the validation cohort, the C-index was 0.772, and the calibration plots showed good probability consistencies between the nomogram prediction and actual observation (Fig. 4E–G).

### Discriminatory powers of the nomogram

**Figure 5:**
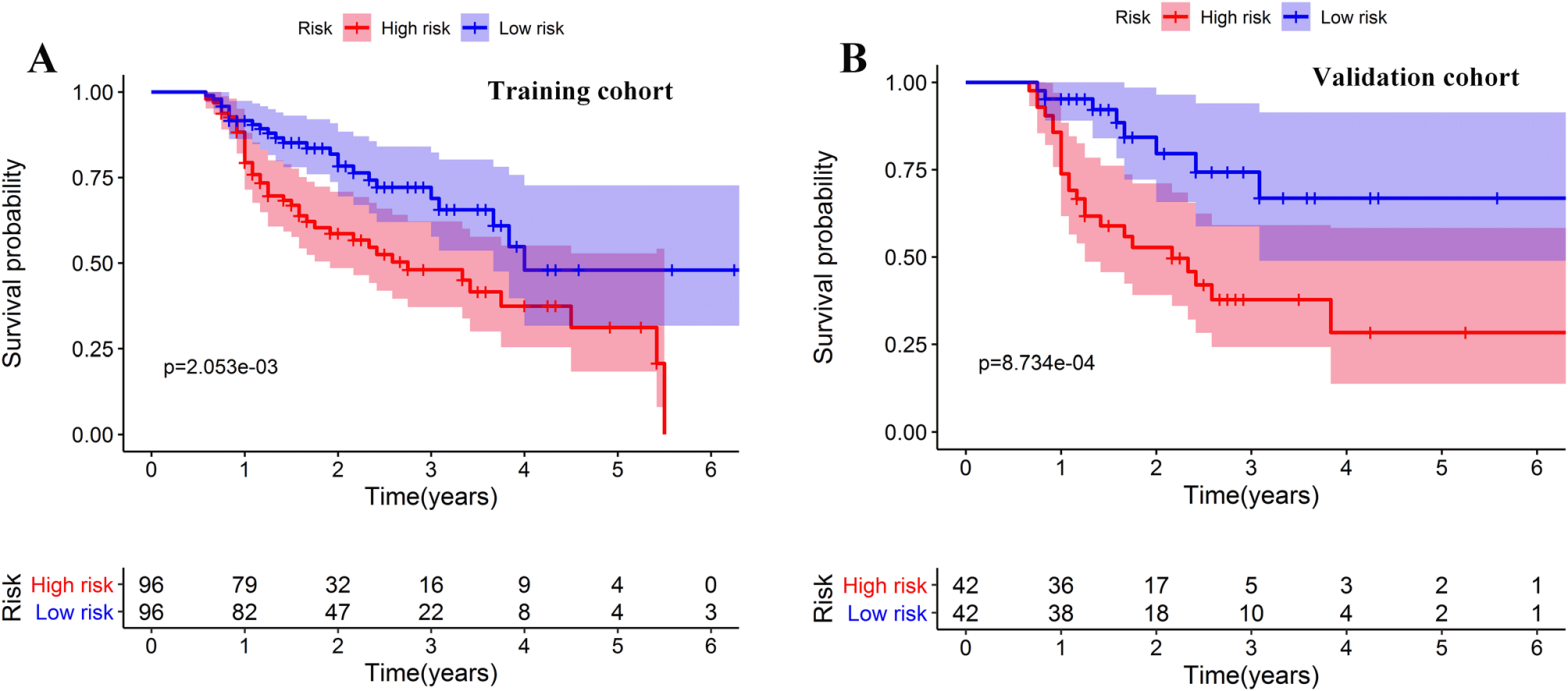
Kaplan–Meier curves of different risk groups stratified by the median value of riskscore calculated by the nomogram in the training **(A)** and validation **(B)** cohorts. The figure was performed using R software (version 3.3.1, Vienna, Austria, https://www.r-project.org/).

Each patient would get a score for risk stratification of death based on the established nomogram. Patients were further divided into high- and low- risk groups based on the median value of riskscore. KM curves and log-rank tests were performed to compare the survival of different groups in the training and validation cohorts. Results showed the constructed nomogram showed clearly different prognostic strata in the training (Fig. 5A) and validation cohorts (Fig. 5B), with both the log-rank *P* values being less than 0.001.

### Comparison of predictive accuracy between nomogram, BCLC staging system and tumor size in samples’ cohort

**Figure 6:**
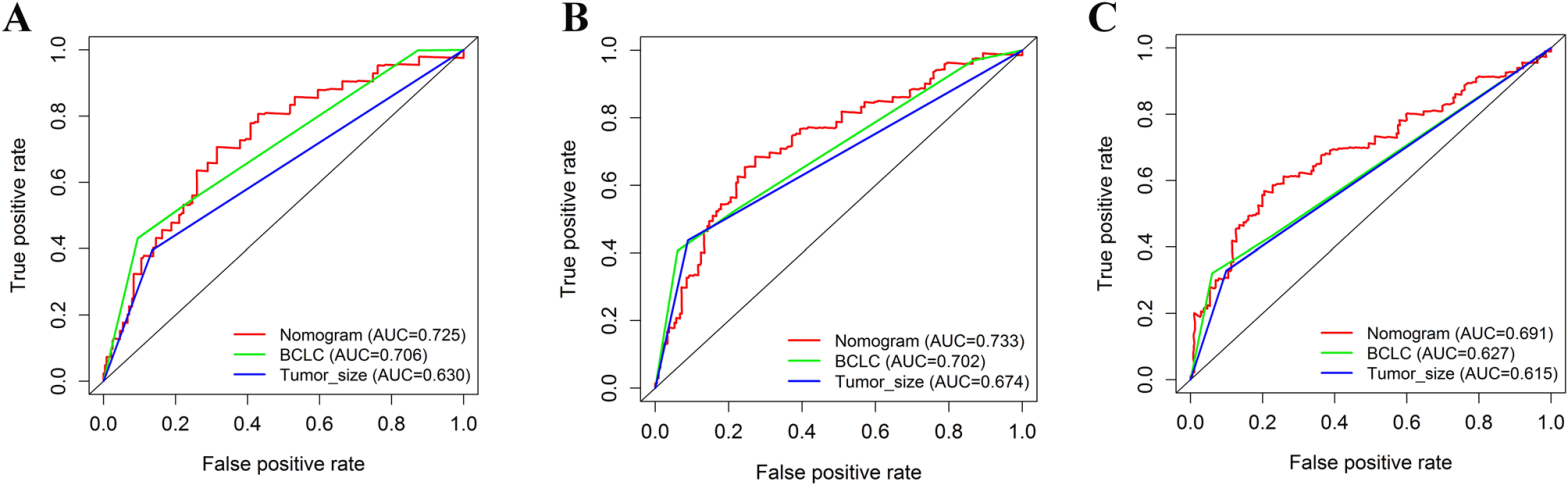
Time-dependent ROC curves for the OS in all samples’ cohort using the constructed nomogram, Barcelona Clinic Liver Cancer (BCLC) staging system and simple tumor size. The area under the curve (AUC) generated from their time-dependent ROC curves for predicting the OS at 1- **(A)**, 3- **(B)**, and 5-year **(C)** was presented. The figure was performed using R software (version 3.3.1, Vienna, Austria, https://www.r-project.org/).

The discrimination ability of the nomogram with BCLC staging system and tumor size were compared in OS prediction. Results from time-dependent ROC curve analyses showed the nomogram displayed better accuracy in predicting OS than BCLC staging system and simple tumor size. The time-dependent AUC of the nomogram for predicting 1-, 3-, and 5-year OS was 0.725, 0.733, and 0.691, respectively. The time-dependent AUCs of the nomogram for predicting 1-, 3-, and 5-year OS were significantly larger than BCLC staging system and simple tumor size (Fig. 6A–C).

### Comparison of predictive power between the nomogram and present models

Subsequently, the predictive power of the constructed nomogram with present models including PNI, King score, GUCI, FIB4, APRI, S-index, and GPR was compared based on C-index. As shown in Table 4, the nomogram discovered the highest diagnostic accuracy in predicting postoperative clinical outcome than BCLC staging system as well as the combination of the present models with tumor size.

**Table 4:** Ranking of discriminatory ability of the prognostic systems on the basis of C-index.

Rank	Systems	C-index	SD	Z
1	Nomogram	0.785	0.046	12.31
2	PNI + tumor size	0.671	0.061	5.64
3	BCLC stage	0.631	0.054	5.61
4	King score + tumor size	0.626	0.064	3.97
5	GUCI + tumor size	0.624	0.064	3.88
6	FIB4 + tumor size	0.617	0.067	3.47
7	APRI + tumor size	0.615	0.066	3.48
8	S-index + tumor size	0.614	0.066	3.44
9	GPR + tumor size	0.611	0.067	3.29

C-index reflects the ability to predict survival: the greater the C-index, the more accurate the prognostic prediction.

*C-index* concordance index, *PNI* prognostic nutritional index, *BCLC* Barcelona clinical liver cancer, *GUCI* Gӧteborg University Cirrhosis Index, *FIB4* fibrosis-4 index, *APRI* aspartate-to-platelet ratio index, *GPR *gamma-glutamyl transpeptidase-to-platelet ratio, *SD* standard deviation.

## Discussion

Despite an improving progress in the multimodal therapy, the survival benefit of HCC patients after surgery remains to be limited. The risk of postoperative HCC early death must be predicted and stratified to allow early intervention for high-risk patients. However, the internationally recognized and widely used systems include the AJCC TNM and BCLC staging systems have not achieved satisfactory results in predicting the prognosis, mainly because these systems simple focus on the tumor clinical characteristics like morphological traits^19^. Clearly, malignant patients’ clinical outcome is determined not solely by tumor characteristics that only reflect the degree of cancer progression, but also by host-related factors such as host response to systemic inflammation^20^. Hence, we expected that a combination of host-related factors with conventional tumor characteristics could accurately predict individualized OS probability in HCC patients after WMA.

Recently, the importance of host inflammatory microenvironment in carcinogenesis, tumor growth, invasion, and metastasis has been recognized^11,21,22^. Better understanding the associations of host systemic inflammation with cancer might contribute to disease prevention and treatment^23^. Host systemic inflammation response could well reflect by changing in circulating white blood cell counts^12^. However, single blood cell count easily be affected by host swollen or dehydrated^24^. Thus, it alone might be difficult to reflect the association of host systemic inflammation with patients’ outcome. Hence, systemic inflammatory indexes including AGR, NLR, LMR, and PLR computed by combination of those cell counts or protein levels have been considered as prognostic indicators in predicting the clinical outcome of patients with malignant tumors.

In the present study, we investigated the prognostic significance of pretreatment AGR, NLR, LMR, and PLR in HCC patients undergoing MWA. Results showed that all the biomarkers were significantly associated with OS. Multivariate analysis further verified that preoperative AGR and PLR were the independent risk biomarker in predicting postoperative OS. In the course of the investigation, we also found biomarkers were significantly associated with advanced tumor and poor host nutrition, which also provided sturdy proof that the progress of the tumor cannot stand away from poor host nutrition and systemic inflammation syndrome^25^.

Although prognostic significance of inflammatory biomarkers has been verified, the underlying mechanism largely remains not be understood. Several following reasons might partly explain the mechanism. Firstly, albumin coming from the liver mainly maintains osmotic pressure and functions as a vector to transport various host metabolic substances. Besides, albumin as an antioxidant has ability to buffer biochemical substance of host internal environment, stabilize cell growth and DNA replication^26^. Albumin not only significantly reflects host nutrition condition, but also associates with host systematically chronic inflammation^27,28^. Hypoalbuminemia could weaken host defense system, thus increasing susceptibility to infection and cytokine^29,30^. Secondly, globulin consisted of all the proinflammatory protein could largely explain host systematic inflammatory response. Inflammation can alter tumor cell biological characteristic and destroy immune function, thus leading to malignancy patients’ poor clinical outcome^31^. HBV infection is associated with HCC occurring and relapsing via mediating HBV immunogenic protein expression, thus resulting in a powerful chronic inflammation^32–35^. Besides, the present study also showed that patients with HBV infection had significantly worse RFS compared with counterparts. Inhibiting inflammatory pathways by regularly taking aspirin could also contribute to better OS and RFS for malignancy patients^36,37^. Thirdly, lymphocyte is a basic component of the cellular basis of immunosurveillance, immunoediting, and innate immune system, which mediates cellular immune reaction to destroy residual cancer cells and micro metastases and then inhibit tumor cells' proliferation and migration^38,39^. Prior study assessed that increasing infiltration of lymphocytes in circulating blood has been statically related to well prognosis^40,41^. Finally, meta-analyses and studies have evaluated the associations of platelet count with malignant tumor^42,43^. Cancer cell could produce a large number of platelet-derived growth factors, which conduce to sustain proliferative signal and promote tumor progression^44,45^.

Recently, nomograms have shown high reliability for predicting tumor progression as a statistical model. Subsequently, we construct a prognostic nomogram for OS in HCC patients after MWA based on those powerful biomarkers and conventional clinicopathological markers including ascites, tumor size, and cancer embolus. Calibration plots identified that nomogram performed well in predicted 1-, 3-, and 5-year OS with ideal model in the training and validation cohorts, which indicated that the nomogram was well calibrated to predict OS in HCC patients after MWA at assessing the performance characteristics. Besides, the nomogram exhibited good efficacy in estimating postoperative OS with a high C-index in the training and validation cohorts. We divided the patients into low-, intermediate-, and high risk groups based on the total score of the nomogram in the training and validation cohorts. Results showed the constructed nomogram showed clearly different prognostic strata in the training and validation cohorts, which confirmed the discriminatory accuracy of the constructed nomogram. According to the nomogram, we can assess the risk of early death of HCC after surgery and conduct early individualized interventions including early postoperative trans arterial chemoembolization (TACE) or postoperative immunoregulatory therapy for high-risk patients. The comparisons of predictive power between our nomograms and the conventional BCLC staging system as well as simple tumor size showed that the constructed nomogram is infinitely superior. Anyway, the nomogram established in the present study showed a robust and remarkably prognostic prediction ability.

The present study might be disturbed by possible limitations as a single center, retrospective, and relatively small-sized sample, which might have had a negative impact on the findings. The findings remain to be confirmed by multicenter prospective clinical studies. In addition, relevant mechanism of systematic inflammatory response to tumor progress was not investigated in the present study. Thus further basic researches need to be done to definite the detailed mechanism. Despite the limitations, the nomogram for predicting survival of HCC patients after MWA showed great discrimination and calibration ability.

## Conclusion

In conclusion, our findings highlighted the prognostic value of systemic inflammatory biomarkers and provide extra evidence for the treatment of HCC and understanding of the possible mechanism of systemic inflammatory reaction in the development and progression of HCC. Furthermore, we established and validated a robust nomogram that could accurately predict individualized survival probability and might support clinician in individual treatment optimization and clinical decision-making for HCC patients after MWA.

## Materials and methods

### Patients

A consecutive set of 327 HCC patients receiving MWA as initial therapy at Jing Zhou Central Hospital, the second clinical medical college, Yangtze University between December 2011 and September 2018 were included in the retrospective study. The following patients were excluded in the analysis: (1) patients with other types of malignancy (n = 2); (2) patients died of non-cancer reason (n = 3); (3) patients with autoimmune disease, systemic infection, or inflammation (n = 5); (4) patients with previous anti-inflammatory medications taken within 1-week (n = 3); (5) patients with severe disease including heart or renal failure (n = 3). (6) patients with incomplete data computed those inflammatory biomarkers (n = 15); (7) patients with incomplete follow-up data (n = 20). Eligible patients met the following criteria: (1) patients with complete peripheral blood cell counts data and follow-up data; (2) patients older than 18-year-old; (3) patients with newly diagnosed by the histological and/or radiology recommended by the America Association for the Study of the Liver Diseases guidelines. Finally, 276 patients remained and were analyzed in this study. Hepatobiliary surgery in our hospital were divided into two independent wards. Hence, 192 patients from the first ward of hepatobiliary surgery were classified as training cohort. Whereas, 84 patients from the second of hepatobiliary surgery were classified as validation cohort. All the HCC patients with HBV or HCV infection are routinely given oral antiviral drugs such as entecavir before surgery until their viral load was below the minimum, and they also regularly take antiviral drugs after surgery in our department. Approval of protocol was obtained from the Ethical Committee of Jing Zhou Central Hospital, the second clinical medical college, Yangtze University. Written informed consent was signed from every eligible patient and their family members. The methods were carried out based on the guidelines and regulations. Patients' records were anonymity and de-identified prior to analysis. The study was carried out in accordance with the Declaration of Helsinki.

### MWA procedure

After routine preparation before the procedure, patients were performed by intravenous anesthesia. Laparoscopic exploration was used to confirm whether there were any other site metastases. Then intraoperative ultrasound was first taken to confirm the puncture path, location, and the number of target lesion. MWA electrode probe was inserted along the path to reach the opposite edge of the tumor lesion through its center. After confirming the location of the MWA electrode probe, MWA treatment was performed. Microwave power was set to 55–70 W and the procedure lasted for 10–20 min. The distance from edge of the ablation zone to tumor edge is larger than 2 cm or very close to the dangerous structure. Subsequently, MWA electrode probe was removed. Finally, intraoperative ultrasound was performed to reevaluate the ablation zone and to determine whether there was another lesion. Laparoscopic exploration confirmed whether serious complications such as bleeding again.

### Follow-up

Routinely follow-up was conducted every 3 months for the first year, every 4 months for the second year, and every 6 months thereafter in our hospital. The detail content contains postoperative AFP, ultrasonic and/or abdominal computed tomography (CT). Follow-up was terminated in October 2019. When serum AFP value more than 20 ng/ml and ultrasonic/abdominal CT finds new focus, patient was considered to tumor recurrence. OS was defined as the interval between surgery and death or the last follow-up. Two clinicians completed follow-up and recorded, respectively.

### Data collection

Baseline clinicopathological variables including age at surgery, gender, demographic data (hypertension, diabetes mellitus, smoking and drinking), tumor size, tumor number, Child‐Pugh class, liver cirrhosis, hepatitis B virus (HBV), hepatitis C virus (HCV), ascites, caner embolus and capsule based radiological or ultrasonic results were obtained. Diabetes was defined as self-reported history of diabetes mellitus, diabetes medication uses or fasting glucose ≥ 126 mg/dl. Hypertension was defined based on a prior diagnosis of hypertension and/or current treatment with medications for hypertension. Whose patient used to smoke or drink and still did not stop at admission was supposed to be a smoker of drinker. Clinical, radiological criteria (computed tomography scan), and histological evidence were used to diagnose the presence of liver cirrhosis in all patients. patients with hepatitis B infection were defined as those who were seropositive for hepatitis B virus surface antigen, and patients with hepatitis C were defined as those who were seropositive for hepatitis C virus antibody in the study. Blood cell counts included neutrophil, lymphocyte, monocyte, platelet, and globulin were extracted from peripheral blood test prior to surgery.

As described previously, those systemic inflammatory markers are computed using the equations as follows: AGR = albumin/globulin, NLR = neutrophil/lymphocyte ratio, LMR = lymphocyte/monocyte, and PLR = platelet/lymphocyte.

### Statistical analysis

The R package of “timeROC” was used to explore the predictive value of systemic inflammatory biomarkers. Based on the median value of systemic inflammatory biomarkers, all the patients were classified into high- and low- risk groups, respectively. (Kaplan–Meier) K-M survival curve was used to compare the prognosis of the high- and low-risk group patients. Whether normal distribution was studied via Kolmogorov–Smirnov test. The relationships of those biomarkers with baseline clinicopathological variables were determined by the nonparametric Wilcoxon rank-sum test or the Kruskal Wallis test. All data were shown as median and range. Univariate Cox proportional hazards model was performed to assess significantly prognostic indicators. Variables showing significantly prognostic value in univariate Cox proportional hazards model were further analyzed in the final multivariate analysis. Based on those independent predictors, a prognostic nomogram was established by R Software version 3.3.1 using the package of rms in the training cohort. ROC curves, Harrell’s concordance indexes (C-index) and calibration plots were used to assess the performance characteristics of nomogram. C-index ranges from 0.5 (no predictive power) to 1 (perfect prediction). The results were externally validated using the validation cohort patients. C-indexes and calibration plots of both the training and validation cohort were based on 1000 bootstrap samples. Hence, each patient would get a score for risk stratification of death based on the established nomogram. Patients were further divided into high-, and low risk groups based on the median value of riskscore. KM curves and log-rank tests were performed to compare the survival of different groups in both cohorts.

Subsequently, the discrimination ability of our nomogram model, Barcelona Clinic Liver Cancer (BCLC) staging system and tumor size in predicting 1-, 3-, and 5- year survival was compared based on time-dependent ROC curves in all samples’ cohort. The corresponding time-dependent area under the curve (AUC) was also computed using the package of “timeROC”. Finally, the predictive performance of the nomogram was compared with the present model including the prognostic nutritional index (PNI)^46^, King score ^47^, Gӧteborg University Cirrhosis Index (GUCI) ^48^, fibrosis-4 index (FIB4)^49^, Aspartate-to-platelet ratio index (APRI)^50^, S-index^51^, and gamma-glutamyl transpeptidase-to-platelet ratio (GPR)^52^. Statistical analyses were performed using SPSS software (version 23.0; IBM Corporation, Armonk, NY, USA) and R software (R Foundation for Statistical Computing, version 3.3.1, Vienna, Austria, http://www.r-project.org/) with packages including "survival", "rms", "foreign", and "survivalROC" “timeROC” and “survminer”. Two-sided P-value < 0.05 was considered statistically significant in all the analyses of the study.

### Ethics declarations

Approval of protocol was obtained from the Ethical Committee of Jing Zhou Central Hospital, the second clinical medical college, Yangtze University. Written informed consent was signed from every eligible patient and their family members. The methods were carried out based on the guidelines and regulations. Patients' records were anonymity and de-identified prior to analysis. The study was carried out in accordance with the Declaration of Helsinki.

## Data Availability

The datasets generated during and/or analyzed during the current study are available from the corresponding author on reasonable request.
